# Alkali Vapor MEMS Cells Technology toward High-Vacuum Self-Pumping MEMS Cell for Atomic Spectroscopy

**DOI:** 10.3390/mi9080405

**Published:** 2018-08-16

**Authors:** Pawel Knapkiewicz

**Affiliations:** Wroclaw University of Science and Technology, Faculty of Microsystem Electronics and Photonics, Janiszewskiego Str. 11/17, 50-372 Wroclaw, Poland; pawel.knapkiewicz@pwr.edu.pl; Tel.: +48-71-320-48-12

**Keywords:** alkali cells, MEMS vapor cells, optical cells, atomic spectroscopy, microtechnology, microfabrication, MEMS

## Abstract

The high-vacuum self-pumping MEMS cell for atomic spectroscopy presented here is the result of the technological achievements of the author and the research group in which he works. A high-temperature anodic bonding process in vacuum or buffer gas atmosphere and the influence of the process on the inner gas composition inside a MEMS structure were studied. A laser-induced alkali vapor introduction method from solid-state pill-like dispenser is presented as well. The technologies mentioned above are groundbreaking achievements that have allowed the building of the first European miniature atomic clock, and they are the basis for other solutions, including high-vacuum optical MEMS. Following description of the key technologies, high-vacuum self-pumping MEMS cell construction and preliminary measurement results are reported. This unique solution makes it possible to achieve a 10^−6^ Torr vacuum level inside the cell in the presence of saturated rubidium vapor, paving the way to building a new class of optical reference cells for atomic spectroscopy. Because the level of vacuum is high enough, experiments with cold atoms are potentially feasible.

## 1. Introduction

The need to develop miniaturized, low power consumption and low-cost instruments/sensors is a current trend. This need is driven by requirements for new applications in which size, weight, and power consumption are key parameters. One example is the development of the miniature, so-called chip-scale atomic clocks (CSAC), applying the Coherent Population Trapping effect (CPT) [1]. The CPT effect is similar to Electromagnetically Induced Transparency (EIT) [2], with the difference being that microwave (electromagnetic) interactions with atoms have been replaced by optical interactions (properly modulated laser light). Using the laser technique, miniaturization of the optical alkali vapor (Cs/Rb) cell and the instrument itself have been made possible. The range of application has expanded as a natural consequence of the availability of small and relatively cheap time and frequency atomic references.

Atomic standards enable very precise control of time and frequency (atomic clocks), as well as high precision of magnetic field measurements. Research on miniature atomic references using the MEMS alkali atom cell is being conducted by several research groups. The common motivation to work on this topic is the growing demand for high-precision and accurate time and frequency references, mostly for telecommunication (terrestrial base stations for telecommunication) and global navigation satellite systems (GNSS), and also for the development of highly sensitive magnetometers. To become competitive solutions for crystal-based time references (TCXO, OCXO), atomic standards must comply with a frequency stability of about × 10^−11^ τ, low power consumption of ~100 mW, size of a few cubic centimeters (~10–30 cm^3^), and be mass producible to give a low price. This is possible through miniaturization and integration using microengineering technology.

The technological development and application extension of atomic time and frequency references has occurred over the last three decades and has resulted in breakthrough discoveries in the field of physics; in particular, optical, laser-based spectroscopy, including the CPT effect mentioned above, and methods for cooling and trapping atoms. The last achievement was awarded the Nobel Prize in 1997 (Steven Chu, Claude Cohen-Tannoudji, William D. P) and was followed by the obtaining of a new state of matter, the so-called Bose-Einstein condensate (Nobel Prize 2001: Eric Cornell, Carl Wieman, Wolfgang Ketterle). Cold atom spectroscopy is most spectacular, due to its future use in the construction of accurate atomic time and frequency standards, and short-term stability (Allan deviation), counted as xE-16, has been impressive. Unfortunately, these solutions exist as laboratory compact setups or laboratory benches only. The key component of such constructions is optical cells, in which 10^−8^ Torr or better vacuum is required inside. Currently, such high vacuum levels can only be obtained by standard pumping systems, because miniature and ready-to-integrate high-vacuum pumping systems do not exist. This is the main reason these solutions have not been miniaturized to date.

Technology of MEMS optical cells for atomic spectroscopy is being developed in several research groups [3,4,5,6,7,8,9,10,11,12], including the one represented by the author [13,14]. The author’s activity in this field started in 2006. During this period, key technologies of miniature, silicon-glass optical MEMS cells, like non-standard anodic bonding sealing processes in buffer gas atmospheres, as well as the novel cesium vapor introduction during laser-induced dispensing from a solid-state dispenser, have been invented [13,14,15] and successfully implemented under the MAC-TFC FP7 Project [16], which has resulted in the first European CSAC.

The purpose of this paper is to present important achievements in MEMS vapor cells and high-vacuum MEMS technology toward the development of self-pumping MEMS cells for atomic spectroscopy. The final achievements have no equivalent at the global scale, and are based on the results of work carried out by the team represented by the author. The experience and knowledge gained so far in tandem with the recently developed MEMS ion-sorption pump [17,18] makes it possible to think that achieving the critical condition of high vacuum inside the cell for the development of MEMS optical cell for cold atom spectroscopy is possible.

## 2. MEMS Alkali Vapor Optical Cell Technology

MEMS alkali vapor cells can be fabricated in several ways [19]. Silicon-glass technology dominates, but low temperature co-fired ceramics (LTCC) ceramic-based solutions have been described in the literature. In this paper, wider analysis of available technologies will not be done. The author will focus on silicon-glass technology, including laser-induced the alkali vapor introduction method, which is crucial for development of the high-vacuum self-pumping MEMS cell.

**Figure 1:**
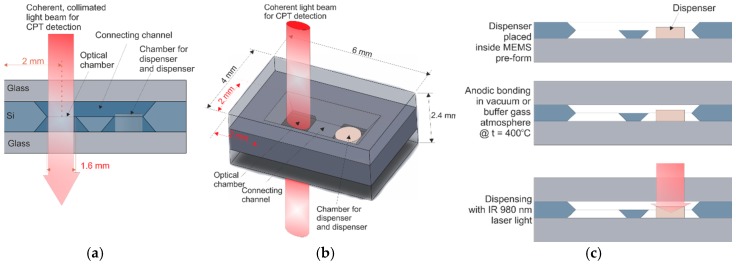
The MEMS cell visualization: (**a**) cross-section view; (**b**) 3D view with dimensions; (**c**) technology path.

The MEMS cell technology involves execution of a miniature hermetically sealed silicon-glass structure. The structure consists of deep reactive ion etching (DRIE), or wet etched silicon body, both sides of which are covered with glass wafers (borosilicate glass). The internal structure is composed of an optical chamber, connection channel and chamber for small, pill-like solid-state cesium dispenser (SAES Getters [20]) (Figure 1a,b).

Silicon wafer with etched cavities is anodically bonded to the bottom glass (>400 °C, 1 kV) to produce MEMS pre-form (Figure 1c). Next, cesium/rubidium dispenser is placed inside the proper cavity. Subsequently, the MEMS pre-form is anodically bonded at approximately 400 °C in vacuum or buffer gas atmosphere to the top side of the glass plate. The applied voltage is 1.5 kV in vacuum or 0.5–1 kV in the presence of buffer gases. The alkali vapor is introduced with the use of NIR laser light (980 nm wavelength) focused onto a pill-like dispenser, where it is absorbed. The dispenser becomes hot and evaporates the alkali vapors, while the rest of the cell remains cold. The intensity and quantity of the introduction of alkali atoms can be set with laser power and irradiation time.

High-temperature anodic bonding (assembling/sealing process) and laser-induced alkali vapor introduction are key methods in silicon-glass MEMS cell technology. Both methods will be described later.

### 2.1. High-Temperature Anodic Bonding and Inner Atmosphere Composition

**Figure 2:**
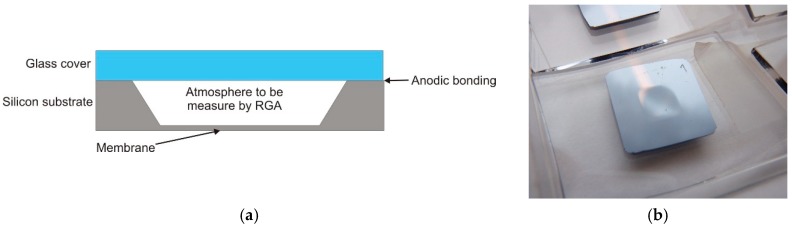
The test structure: (**a**) schematics of the cross-section; (**b**) real view of the structure where the deflected membrane is visible.

Out-gassing of residual gasses (or particles) from the inner cell walls from the bonded interface might contaminate the inner atmosphere of the MEMS cell and influence the optical properties and long-term stability of the cell. To confirm the quality of anodic bonding, and to obtain information on the atmosphere composition of cells sealed by anodic bonding, several test samples were fabricated, followed by Residual Gas Analysis (RGA). The test samples consist of a silicon substrate with a deep wet-etched cavity forming a large-area Si membrane (5 × 5 mm^2^) and a glass cover (Figure 2).

Silicon substrate and glass cover were bonded using the anodic bonding process in vacuum with buffer gas (argon) atmosphere as described above. Part of the residual gases—present inside the chamber used for anodic bonding—and the buffer gas were trapped inside the test samples. As is shown in Figure 2b, the deflection of the membrane was visible to the naked eye on the test samples after the anodic bonding process, due to the lower Ar pressure inside the cells compared to the outside atmospheric pressure.

**Figure 3:**
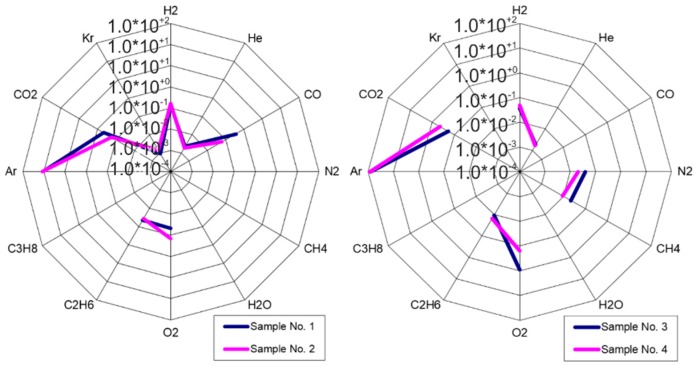
Star-like graphs showing high repeatability of proposed sealing process.

RGA analysis results (Table 1) obtained before or after aging (twin cells, fabricated in the same process, were used; the aging procedure is described later), show that the sealing process—using a modified cleaning and hydrophilization procedure prior to the high-temperature anodic bonding process—gives good results. The composition of the inner atmosphere is stable and shows good repeatability (Figure 3). Low amounts of contaminations (O_2_, CO, CO_2_, CH_4_, C_2_H_6_) are characteristic for vacuum MEMS structures sealed with the anodic bonding process.

Additionally, analysis of residual gas composition inside the silicon-glass structures after anodic bonding was performed at 2 × 10^−3^ mbar with and without MEMS getter (SAES Getters, Italy) (Table 2). Results obtained for samples without getter are similar to those presented before. Inner atmosphere inside the test structure with MEMS getter is clean, except for a low (10^−5^ mbar) amount of argon. The internal atmosphere is almost entirely filled with helium (10^−3^ mbar). This vacuum level is the minimum to obtain with the use of MEMS getter.

The experiment related to the high-temperature anodic bonding and the study of the inner atmosphere revealed the following important facts:

- The inner atmosphere of the MEMS structure is contaminated by products of the anodic bonding process (O_2_, CO, CO_2_, CH_4_, C_2_H_6_), resulting in a vacuum no better than 10^−1^ mbar,
- Contaminations can be removed from the inner atmosphere with use of MEMS getters, except noble gases, especially helium, whose pressure was maintained at 1 × 10^−3^ mbar,
- Preprocessing (wafers cleaning, hydrophilization) followed by high-temperature anodic bonding minimizes problems related to out gassing and gas penetration through the bonded silicon-glass interface, which has previously been visible as an unnoticeable effect of the structure’s aging.

The obtained results are sufficient for atomic clock technology (CSAC) using the CPT effect. A vacuum level of 1 ×10^−3^ mbar is far from the value required to cool the atoms, but is a good starting point for a MEMS ion-sorption pump, for which integration with the MEMS alkali vapor optical cell is planned.

### 2.2. Laser-Induced Alkali Vapor Introduction Method from Solid-State Dispenser

Alkali vapor dispensers are available as resistively heated wires. This solution has been known for years and is still in use. Forming the dispenser into a pill and using it in the MEMS optical cell technology has been invented by author’s group and successfully implemented in the MAC-TFC FP6 Project. The purpose of the Project was the development of the first European miniature atomic clock, what was achieved [16]. The laser-induced cesium vapor introduction method from solid-state dispensers is the basis of the technology of MEMS optical cells developed in the author’s mother’s unit, but also at the Université de Franche-Comté/FEMTO-ST, Besancon, France [21,22].

To evacuate liquid alkali and their vapor effectively, the dispenser must be heated to above 650 °C. The 980 nm and 4 W of maximum power (continues work mode) IR laser-based activation set-up was built. The mechanical part of the set-up ensured precision positioning of a cell. The electronic part ensured precision power control of the IR beam, activation time setting and their automatic timing, and real-time viewing, as well as video recording of the activation process. Effective introduction of alkali vapors depends on the cell design and inner atmosphere conditions. Distance to walls and the presence of buffer gas change heat dissipation, due to the fact that the laser power and irradiation time must be set individually.

### 2.3. Accelerating Aging Tests of MEMS Cesium Cells

**Figure 4:**
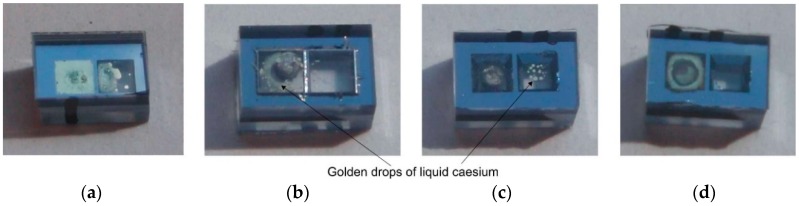
Four examples of MEMS cesium cells fabricated at Wroclaw University of Technology: (**a**) 50 mbar of Ar—large amount of alkali atoms, (**b**) 100 mbar of Ar—evaporated metal located on dispenser’s chamber, (**c**) 200 mbar of Ar—small amount of cesium in form of golden drops condensate in optical chamber, (**d**) 300 mbar of Ar—alkali metal visible as golden fog around dispenser.

Several MEMS cesium cells of 1.0 mm optical path and 4 × 6 mm^2^ planar dimensions (see Figure 1a,b), filled with Argon as buffer gas with different pressures in the range from 50 mbar to 300 mbar, were successfully fabricated (Figure 4). MEMS getters were intentionally not applied, to check for the influence of residual gases on inner atmosphere quality.

Alkali vapor dispensing was carried out for fixed values of IR laser power (~0.8 W) and irradiation time (15 s). Different amounts of alkali metal were released depending on the pressure of the buffering gas. Increasing pressure improves heat dissipation; thus, less power is supplied to the dispenser. However, this has positive sides in the form of better control of the amount of released atoms.

All MEMS cells were exposed to accelerated aging tests. There are no existing norms on aging test procedures for miniature MEMS alkali vapor cells. The known aging procedures for electronic devices/components are focused on generation of mechanical tensions as result of temperature changes (temperature cycles). The goal of aging of MEMS cesium cells is to see possible degradation of alkali metal (oxidation—cesium becomes black) caused by contaminations out gassed from bulk materials (mostly glass) and the bonding interface. Out gassing is a function of temperature. We propose a new temperature-accelerated aging procedure for miniature MEMS alkali vapor cells, based on a simplified model for the cell aging. The acceleration factor (AF) is a function of ΔT = TX − TN, where TN is the nominal operating temperature, and TX the aging temperature:

AF = 2^0.1 × ΔT^ (1)

**Figure 5:**
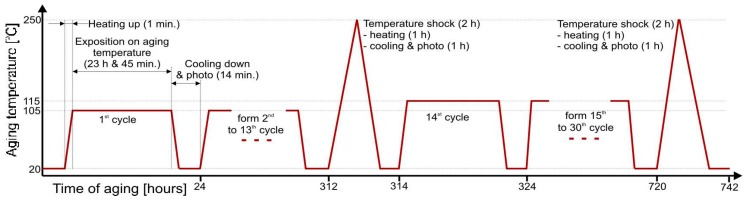
The temperature profile of the accelerated aging test.

The operating temperature of the MEMS cell should be kept in the range from 40 °C to 85 °C. The normal operating temperature has been set to TN = 75 °C. The applied temperature profile is presented in Figure 5 and consists of cycles with 24 h periods. Each 24 h cycle consists of three steps: heating up the cell from ambient temperature to aging temperature (105 °C or 115 °C) in 1 min, exposition at aging temperature for 23 h and 45 min, cooling down to ambient temperature to make optical observations and pictures—15 min. The cells underwent 13 cycles, i.e., a total of 309 h at TX = 105 °C (corresponding to 2472 h at 75 °C), followed by 16 cycles (380 h) at TX = 115 °C (corresponding to 6080 h at 75 °C). Moreover, after aging at 105 °C and 115 °C, respectively, short temperature shocks were applied where the temperature reaches 250 °C.

During the test, no changes in the amount or color of the cesium drops (as an indication of possible oxidation) were observed. This indicates that the inner atmosphere is clean and stable. The explanation is simple. The inner atmosphere of the cell consists of saturated cesium vapors. Cesium, as a highly reactive atom, reacts with residual gases and neutralizes them. Even if some part of cesium atoms reacts with impurities, the saturation of the cesium vapors is still maintained.

The cesium pill-like dispenser composition contains porous alloys or powder mixtures of Al, Zr, Ti, V or Fe. Those materials are the basis of NEG (Non Evaporable Getters). NEG getters are thermally activated (>200 °C). Therefore, at elevated temperatures (laser-induced dispensing, aging tests) the pill-like dispenser plays a dual role as alkali atom dispenser and NEG getter.

Moreover, the high quality and stability of the applied anodic bonding as a sealing process is confirmed by the resistance of the cells to temperature shocks (250 °C) and possible cesium penetration into the bonded interface. Cesium, as a highly reactive atom, may neutralize impurities, but may also degrade materials and the bonded interface. In the tested MEMS cells, the distance between the cell’s inner chamber and the environment was only 1 mm at the narrowest point of the bonded interface. Interface degradation, manifesting as alkali atom oxidation as a result of the atoms’ penetration through interface, was not observed. Golden drops/fog of cesium atoms were always visible.

The high-temperature anodic bonding in vacuum or buffer gas atmosphere described here, as well as the investigation of the atmosphere composition inside the MEMS structures and the laser-induced alkali vapor introduction method, are breakthrough technologies for the development of MEMS optical cells for miniature atomic clocks, but they also stand behind the development of future solutions including high-vacuum MEMS. The most important outcomes from the current work are:

- High-temperature anodic bonding provides durable and tight connection of silicon-glass substrates,
- It is possible to obtain a medium vacuum level (10^−1^–10^−3^) inside the MEMS structures, which gives a good starting point for the MEMS ion-sorption pump,
- The presence of buffering gases favors better control of laser-induced alkali atom introduction; hence, one should pay particular attention to the process of dispensing in high vacuum.

## 3. Self-Pumping MEMS Optical Cell for Atomic Spectroscopy

Atomic spectroscopy—cold atoms spectroscopy, for example—requires at least 10^−8^ Torr vacuum and a low concentration of atoms (partial pressure lower than the vapor pressure). This state of the inner atmosphere can only be achieved in a dynamic manner. This means that maintaining high vacuum and dispensing must be done at the same time.

**Figure 6:**
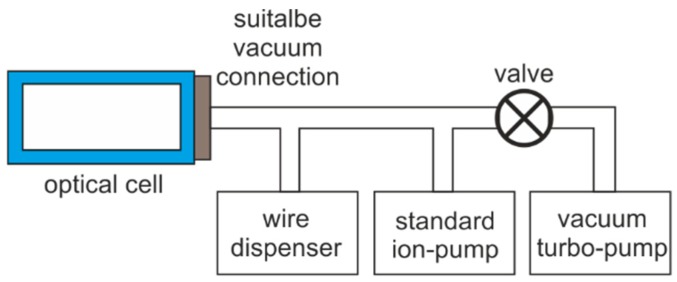
Block diagram of standard setup for atomic spectroscopy at high vacuum and low concentration of atoms.

The standard setup consists of an open-sided cell connected to a typical vacuum installation. The inner atmosphere is firstly evacuated with use of turbo-molecular pump (Figure 6). After achieving 10^−6^ Torr, the turbo pump is disconnected and the ion pump starts work. The alkali atoms are delivered continuously from the wire dispenser through thermally activated chemical reaction (current flowing through the dispenser heats it up). To achieve the proper amount of atoms and the required vacuum level, the ion pump must be running in conjunction with dispensing efficiency.

Development of miniature, integrated MEMS cells for atomic spectroscopy required miniaturization and integration of the pumping system, a proper alkali atoms introduction method, and suitable assembly processes.

The high-temperature anodic bonding described earlier will be used as an assembling method, along with the alkali atoms introduction method from a solid-state pill-like dispenser. Development of integrated, miniaturized pumping system that is suitable for MEMS technology remains a challenge.

The team represented by the author has been conducting research on vacuum microelectronics for many years. This research has resulted in the development of a miniature ion-sorption vacuum pump. The pump makes possible the generation and stabilization of pressure at a 10^−8^ Torr vacuum level, and its construction is fully compatible with MEMS technology. These advantages make the pump usable in new applications [23,24,25] including self-pumping MEMS optical cells for atomic spectroscopy.

**Figure 7:**
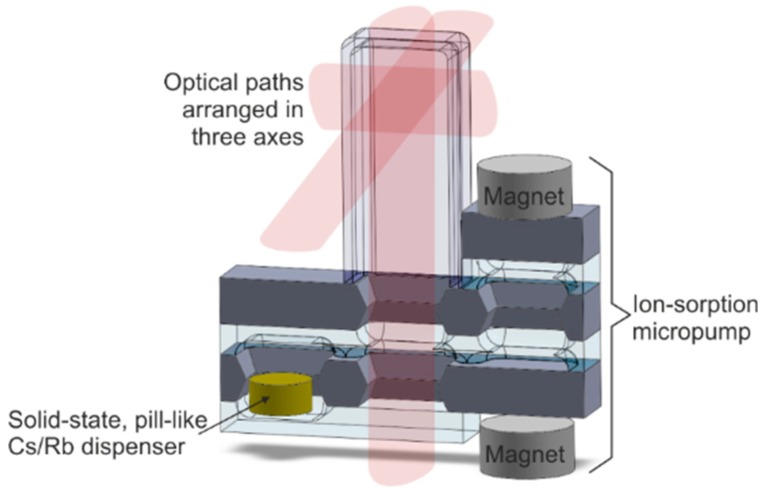
Self-pumping MEMS optical cell for atomic spectroscopy—visualization.

The multilayer silicon-glass spatial structure, consisting of a glass tube with appropriate dimensions connected to a planar structure containing solid-state dispenser and miniaturized ion-sorption pump is proposed (Figure 7). The cell is made of borosilicate (Pyrex-like) glass and silicon. All connections are made with the use of a high-temperature anodic bonding process, which ensures tight and stable leak-proof connections.

Absorption spectroscopy and cold atom spectroscopy were planned using a self-pumping MEMS cell. Because the author has access to the rubidium atomic spectroscopy laboratory, the dispenser was changed to rubidium. This change has no influence on the cell technology.

**Figure 8:**
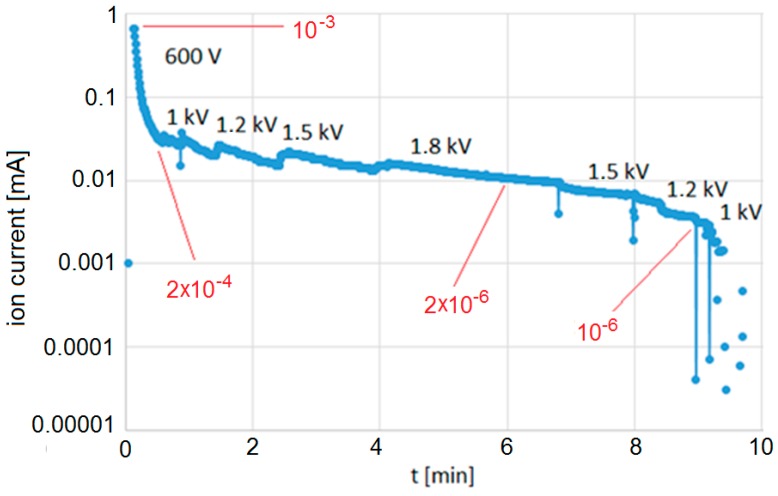
Pumping efficiency in time; vacuum level marked in red.

A self-pumping MEMS cell filled with rubidium vapor was fabricated. First of all, the open test structure of the self-pumping cell was closed inside the vacuum chamber, and ion current versus pressure was measured. Based on the scaling, the initial vacuum and pumping efficiency was measured (Figure 8).

Initial pressure is equal to 10^−3^ Torr just after the sealing process. After a few minutes of pumping, the vacuum reaches 10^−6^ Torr. Vacuum level was improved by increasing the applied voltage and extending the pumping time. Based on the measured ion current, the vacuum level was at least two orders of magnitude better (blue dots on the graph, Figure 8). Because of the pumping limit of the apparatus used for scaling, precise numbers cannot be given.

After pumping, rubidium atoms were introduced through laser-assisted dispensing from a pill-like solid-state dispenser, reaching saturation of the alkali vapor. A more detailed description of vacuum generation and stabilization can be found in [26].

**Figure 9:**
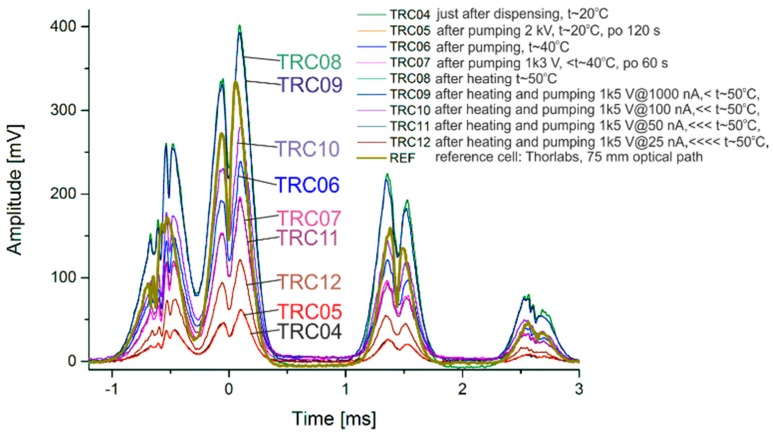
Doppler-free characteristics of the self-pumped rubidium MEMS cell at different test periods.

Doppler-free spectroscopy of rubidium self-pumping MEMS cells was done (D2 line, 780 nm). Characteristics were measured at different test periods (Figure 9). Peak contrast (amplitude) depends on the temperature, and remains comparable to the characteristics of the reference cell. Impurities where partial pressure is higher than 10^−3^ may have an influence on optical spectroscopy. In our experiment, the characteristics are stable: no peak shifts or half-width peaks were observed.

## 4. Summary

The solution presented here is the result of work carried out by the team in which the author is the core member developing the technology described in this article. The description of the construction and initial measurement results of the self-pumping high-vacuum cell was preceded by the explanation of key technologies developed earlier for the needs of the European miniature atomic clock. High-temperature anodic bonding and its influence on the atmosphere composition inside MEMS structure were studied, followed by performing accelerated aging tests of MEMS cesium vapor optical cells. It was found that the sealing process ensured high and stable connection. RGA analysis of the inner atmosphere gives important information. After the sealing process, the inner volume of the MEMS structure is depolluted by chemical products of the anodic bonding process (O_2_, CO, CO_2_, CH_4_, C_2_H_6_), setting the vacuum level at 10^−1^ mbar, even if the bonding process was carried out in a vacuum two orders of magnitude better. MEMS getters improve the situation by eliminating pollution, with the exception of noble gases, whose pressure inside the MEMS structure is set at 10^−3^ mbar, which is the limit using passive vacuum stabilization methods.

The aim of this work is to develop a MEMS optical cell in which it will be possible to generate and stabilize a high vacuum with the simultaneous presence of a small amount of alkaline atoms. Generation of high vacuum inside MEMS structures is possible only with use of active pumping. Integration of the currently developed MEMS ion-sorption pump and MEMS alkali vapor optical cell was proposed, toward a self-pumping, high-vacuum, MEMS alkali vapor optical cell for atomic spectroscopy. The self-pumping high-vacuum cell has no equivalent at a global scale and is probably presented here for the first time ever. High pumping efficiency, making it possible to achieve a 10^−6^ Torr vacuum level, was proved. It must be strongly pointed out here that pumping out of impurities was possible in the presence of saturated rubidium vapor. The presence of saturated alkali atom vapor limited the vacuum level. However, this result makes it possible to think that the generation of a vacuum level of 10^−8^ Torr and the stabilization of partial vapor pressure below saturation is possible.

Doppler-free spectroscopy shows that absorption peaks are comparable to the reference spectrum. Simple absorption tests will not reveal the potential of this new solution. More sophisticated experiments like cold atom spectroscopy, where high vacuum and low concertation of atoms are required, should demonstrate the true possibilities of this solution.

## Figures and Tables

**Table 1:** Residual Gas Analysis (RGA) of atmosphere composition inside test structures before and after aging: table of partial pressure of different particles.

Gas	Sample No. 1 (mbar)	Sample No. 2 (mbar)	Sample No. 3 (mbar)	Sample No. 4 (mbar)
H_2_	1.41 × 10^−1^	1.68 × 10^−1^	3.75 × 10^−2^	4.78 × 10^−2^
He	2.30 × 10^−3^	1.96 × 10^−3^	1.86 × 10^−3^	1.68 × 10^−3^
CO	3.60 × 10^−1^	6.15 × 10^−2^	0.00	0.00
N_2_	0.00	0.00	3.86 × 10^−2^	2.07 × 10^−2^
CH_4_	2.35 × 10^−2^	2.27 × 10^−2^	2.18 × 10^−2^	9.31 × 10^−3^
H_2_O	0.00	0.00	0.00	0.00
O_2_	4.83 × 10^−2^	1.43 × 10^−1^	8.95 × 10^−1^	1.59 × 10^−1^
C_2_H_6_	4.44 × 10^−2^	3.64 × 10^−2^	1.07 × 10^−2^	1.56 × 10^−2^
C_3_H_8_	0.00	0.00	0.00	0.00
Ar	1.11 × 10^+2^	1.16 × 10^+2^	8.36 × 10^+1^	8.77 × 10^+1^
CO_2_	4.55 × 10^−1^	1.75 × 10^−1^	1.87 × *10^−1^	4.35 × 10^−1^
Kr	9.80 × 10^−4^	1.48 × 10^−3^	0.00	0.00
TOT.	1.12 × 10^+2^	1.17 × 10^+2^	8.48 × 10^+1^	8.84 × 10^+1^

**Table 2:** Residual Gas Analysis (RGA) of atmosphere composition inside test structures with and without MEMS getter inside.

Gas	Without Getter		With Getter	
	mbar	%	mbar	%
H_2_	9.24 × 10^−1^	69.72	0.00	0.00
He	8.55 × 10^−4^	0.06	1.06 × 10^−3^	96.70
CO	1.89 × 10^−1^	14.26	0.00	0.00
N2	2.57 × 10^−2^	1.94	0.00	0.00
CH_4_	3.23 × 10^−2^	2.44	0.00	0.00
H_2_O	2.15 × 10^−3^	0.16	0.00	0.00
O_2_	3.88 × 10^−5^	0.00	0.00	0.00
C2H_6_	0.00	0.00	0.00	0.00
C_3_H_8_	0.00	0.00	0.00	0.00
Ar	4.24 × 10^−5^	0.00	3.60 × 10^−5^	3.30
CO_2_	1.51 × 10^−1^	11.41	0.00	0.00
Kr	0.00	0.00	0.00	0.00
TOT.	1.32	100.00	1.09 × 10^−3^	100.00
